# Contextual Variability in Personality From Significant–Other Knowledge and Relational Selves

**DOI:** 10.3389/fpsyg.2015.01882

**Published:** 2016-01-07

**Authors:** Susan M. Andersen, Rugile Tuskeviciute, Elizabeth Przybylinski, Janet N. Ahn, Joy H. Xu

**Affiliations:** Department of Psychology, New York University, New YorkNY, USA

**Keywords:** significant others, relational self, close relationships, transference, cross-situational inconsistency

## Abstract

We argue that the self is intrinsically embedded in an interpersonal context such that it varies in IF–THEN terms, as the *relational self.* We have demonstrated that representations of the significant other and the relationship with that other are automatically activated by situational cues and that this activation affects both experienced and expressed aspects of the self and personality. Here, we expand on developments of the IF–THEN cognitive-affective framework of personality system (Mischel and Shoda, 1995), by extending it to the domain of interpersonal relationships at the dyadic level (Andersen and Chen, 2002). Going beyond Mischel’s early research (Mischel, 1968), our framework combines social cognition and learning theory with a learning-based psychodynamic approach, which provides the basis for extensive research on the social-cognitive process of transference and the relational self as it arises in everyday social interactions (Andersen and Cole, 1990), evidence from which contributes to a modern conceptualization of personality that emphasizes the centrality of the situation.

## Introduction

The notion that people’s responses and behavior will tend to vary by the situation they are in, as a function of internal states, mental representations, and interpretations that are brought to the fore by cues in the situation (Mischel, 1968, 1973, 1977; Wright and Mischel, 1987; Shoda et al., 1994), was iconoclastic when proposed, but is now supported by considerable evidence. While it may seem that people tend to possess global traits that do not vary appreciably by situation, this concept does not do justice to the complex nature of personality. *In situ* research has demonstrated substantial variability in behavior across situations (Mischel, 1968), while stability can be observed in the pattern of behavior individuals engage in across different situations over time (Mischel and Peake, 1982; Mischel and Shoda, 1995). What arises is a kind of *personality signature* or behavior profile across situations. Indeed, variability across situations that is stable over time is now rather widely accepted (e.g., Kenrick and Funder, 1988; Fleeson, 2001; Funder, 2008; Fleeson and Noftle, 2009).

In this article, we present a conceptual framework and a line of research in the interpersonal domain that characterizes individual behavior as the result of context-specific cues and makes use of long-term memory storage in a dynamic way—that is, emphasizing both what the individual brings to the table from personal experience and the situational cues that trigger such experience. We present the social-cognitive model of transference and the relational self, and the research that supports it (e.g., Chen and Andersen, 1999; Andersen and Chen, 2002), as an IF–THEN person-situation interaction model and an interpersonal version of what is known as the cognitive-affective personality system or cognitive-affective processing system (CAPS) approach. Beyond this, we go further here than elsewhere in specifying the relation of our framework to the CAPS model, and further, address explicitly the voluminous literature on trait dispositions (particularly interpersonal traits) and their potential interface with this framework.

The CAPS framework relies on cognitive-affective units (CAUs), which represent individual experience and contribute to an individual’s interpretations and behavior in particular contexts (Mischel and Shoda, 1995; see also Metcalfe and Mischel, 1999). Past experience alters the meaning and significance accorded to present situations, and importantly, the strength and likelihood of relevant behaviors being enacted in those situations. Of central importance in predicting human behavior, and if desired, in changing it, is accounting for the stimuli in situations that prompt particular behavioral patterns (Metcalfe and Mischel, 1999). The cognitive-affective systems model of personality is thus an IF–THEN theory in which the situation—or set of triggering cues—interacts with whatever disposition or set of associations the individual has with these cues, which in turn, places the individual in a distinct psychological situation. Indeed, the situational IF cue(s) evokes a contextual THEN, or the relevant experience and behavior.

In our research, we have examined how mental representations of significant others—that is, any important person, such as a close friend, current or past romantic partner, sibling, or parent whom the individual knows well and has had a considerable impact on the individual—arise as a function of contextual cues, and influence moment-to-moment interpersonal responses on the basis of their implicit activation. Because significant-other representations are often evoked and used, they tend to be chronically accessible (Andersen et al., 1995) and are even more likely to be evoked if triggering cues are present in the situation (Andersen et al., 1995; Chen et al., 1999; see Higgins, 1989, 1990). Hence, in the process known as *transference*, certain cues in a new person, such as his or her behavior or conveyed beliefs, attributes, or even facial features, can activate a relevant significant-other representation. The representation is then applied to understanding the new person. Of course, significant others are, by definition, people in whom the individual is invested emotionally and motivationally (Higgins, 1987, 1997; Hinkley and Andersen, 1996; Andersen et al., 1998). Hence, they allow for special relevance to be accorded to a new person, when triggered in transference, leading the new person to be seen, interpreted, and remembered in terms of significant-other knowledge, while also evoking a variety of relationship-specific and self-with-other experiences that are emotional and motivational in nature.

Accordingly, cues of any subtle resemblance to a significant other in a new person will evoke the significant-other representation, the relational self, and the transference process in IF–THEN terms. In many instances, this process can be interpersonally useful, easing social interactions, and prompting the individual to give new persons the benefit of the doubt, as positive feelings toward the significant other are felt anew toward (“transferred” to) these new persons (e.g., Andersen and Chen, 2002). In fact, under some circumstances, it can even diminish intergroup bias (Saribay and Andersen, 2007) and promote a sense of shared reality (Przybylinski and Andersen, 2012, 2015). However, it can have detrimental consequences as well (e.g., Berenson and Andersen, 2006; Reznik and Andersen, 2007; Berk and Andersen, 2008; Miranda et al., 2013) if the relationship with the activated significant other happens to be troubled in some way, even if this person is otherwise loved.

Given that the concept of transference derives from psychoanalytic thought (Freud, 1958, 1963) as modernized in neo-Freudian and interpersonal terms (Sullivan, 1953), it is perhaps less surprising that we conceptualize and examine it in social-cognitive terms (Andersen and Glassman, 1996; Chen and Andersen, 1999; Andersen and Chen, 2002). Drawing on a set of century-old assumptions about personality, psychological disturbance, and treatment, this work is clearly relevant to bridging the gap between contemporary social cognition, interpersonal approaches to the self, and psychodynamics. Moreover, it construes person-situation interactions as an interpersonal version of a cognitive-affective system approach.

A central contribution of this research is that it begins to populate the CAPS model with needed content and content specificity. Indeed, the CAPS model focuses primarily on process and to a degree, structure, while providing relatively little guidance as to the content of CAUs within the model. The current framework and research does this with a focus on the interpersonal domain, and outlines situations and processes more specifically. In particular, we emphasize how individual behavior varies across interpersonal situations, in a manner determined partly by the process of transference. In the process, elements of the interpersonal situation (i.e., of the person one is interacting with) resemble and, in turn, implicitly and automatically bring to mind a prior significant other, which influences the individual’s inferences about and evaluation of the new person. Indirectly, this activation also brings to mind the relationship with this significant other, as well as the individual’s view of the self in this relationship at the moment, his or her motivation, goals, and regulatory strategies, not to mention emotions and behaviors. Accordingly, in the CAPS model, the CAUs, such as expectancies, goals, affect, and self-regulatory plans, form a “system” of units that interact with each other in mediating behavior. In our approach, these units consist of the various aspects of an individual’s significant-other relationships, and such units are organized in terms of individual representations of specific significant others and one’s relationship with each, which are all stored in memory. Each one can be triggered from memory when the significant other is implicitly activated by subtly relevant cues in the environment (a person, a situation), leading to shifts in observed responses.

Further, our approach treats “dispositions” as reflected and embodied by the content of the longstanding significant-other representations and relationships in memory, which enable both stability in the individual’s responses and the variability that arises in them across relevant interpersonal situations. Research on trait approaches to individual differences defines personality in terms of global dispositions that are shared and nomothetic (people differ in the degree to which they hold a trait, rather than in its qualitative definition), and some trait dispositions are explicitly interpersonal (e.g., need for affiliation, extraversion, agreeableness, dominance). Our approach, by contrast, defines personality and individual differences particularly ideographically, based on prior learning and prior relationship experience. Although we do not argue that all variability across situations in individual responding (or stability over time) is reducible to interpersonal experience alone, nor that significant-other representations and relationships are the sole basis for the content of self and personality, we do simply argue that such knowledge in memory captures meaningful, longstanding, personally relevant knowledge, that anchors the individual in his or her own prior learning and experience, while still enabling variability in individual behavior to emerge as a function of variability in interpersonal contexts.

## The Relational Self

Imagine that a new employee is hired at your workplace. He loves reading mystery novels, much as your adored older brother does, has a similar liking for Italian food, and even similar quirks (e.g., the same bombastic laugh). You immediately like him without knowing why and find yourself holding his opinion in especially high esteem. You even doubt yourself when you disagree with him, which you do not do with other coworkers. In this case, your self-doubt cannot be explained solely by a general personality trait (e.g., insecurity), or by the situation itself (being at work). Our model of transference, however, provides a framework for understanding why, when, and how this specific kind of response happens.

In transference, the representation of a significant other (e.g., one’s brother) will be activated when a new person (e.g., the coworker) resembles that significant other in some subtle way (e.g., has a similar laugh). This resemblance can come in the form of the new person’s personal characteristics, such as interests, behavioral tendencies, values, interpersonal style, specific expressions, or physical appearance. Once the significant-other representation is activated, it tends to be applied to the new person, influencing one’s perception of the new person and one’s responses to him or her (Andersen and Cole, 1990; Andersen and Baum, 1994). Thus, significant-other cues encountered in a situation combined with knowledge stored in memory (which is chronically accessible) about the significant other, affect both interpretations of the new person as well as one’s responses to him or her. For instance, knowledge one has of the significant other is then assumed to be true of the new person who resembles this significant other, in addition to what one actually sees and learns about the new person. The individual then thinks that he or she “learned” this transferred information about the new person, when, in fact, the individual did not.

This effect on memory can be evoked based not only on cues to a new person’s characteristics, but, as implied, also on his or her facial resemblance to a significant other (Kraus and Chen, 2010), and has been shown to persist for weeks (Glassman and Andersen, 1999b). Moreover, cues of either sort can provoke a relatively automatic positive evaluation of a new person when he or she implicitly resembles a significant other who is also regarded positively—that is, liked or loved (e.g., Andersen and Cole, 1990; Andersen and Baum, 1994; Andersen et al., 1995; Chen et al., 1999; Günaydin et al., 2012). Finally, this transference process not only occurs implicitly (Andersen et al., 2005), but can also be triggered by cues presented entirely outside of awareness (Glassman and Andersen, 1999a). The latter is of importance both because the notion of the unconscious is so predominant in psychodynamic theory and in the transference concept, and because it suggests that the process of transference may not be readily detected or intentionally controlled.

Significant-other representations are linked in memory to representations of the self by the relationship with each significant other (Andersen and Chen, 2002). Thus, individuals have a specific relational self associated with each significant other represented in memory (Andersen et al., 1997; Chen and Andersen, 1999; Andersen and Chen, 2002: see also Baldwin, 1992; Chen et al., 2006), reflecting the version of the self generally experienced in that relationship. Accordingly, these versions of the self are also indirectly activated when a significant-other representation is activated as a function of situational triggering cues. Because any significant-other cue can activate the significant-other representation, these cues will also indirectly activate the self-with-significant-other representation and the significant-other relationship. Once these representations are activated, one “becomes” who one typically is with that significant other. Furthermore, motivations and goals relevant to the significant-other relationship are also activated in response to the new person—for instance, one might be particularly motivated to not be candid with him or her. In transference, information about the significant other’s past acceptance or rejection stored in memory should also be activated when the significant-other representation is activated and thus should also be anticipated from the new person. In this way, the significant other need not be physically present to greatly influence the self and interpersonal interactions.

Said differently, significant others have been shown to be represented in memory in a manner that is rich in features and highly distinctive (Andersen and Cole, 1990; Andersen et al., 1998), both in terms of personality characteristics and physical features, as well as in interpersonal styles, habits, and interpersonal tendencies. Moreover, included in such significant-other knowledge are complex IF–THEN units that reflect the particular psychological (internal) states these others experience and how they behave based on them (as situational contexts, Idson and Mischel, 2001; Chen, 2003). Hence, such knowledge structures are complex.

## Relevant Conceptions of Personality

### Traits as Dispositions

Although most trait theorists, historically, have acknowledged the relevance of situations to trait expression (e.g., Allport, 1937; Murray, 1938; Cattell, 1965), and the interaction between the person and the situation, this has not tended to be emphasized or commonly examined in empirical research. That is, the consensus was (and largely is) that trait dispositions are stable over time, as are their correlates, and as such, are worthy of study in their own right, independent of context. This makes sense, and of course, Mischel’s early work also prompted systematic research pitting the person against the situation (and *vice versa)* in numerous trait-situation studies at the debate’s inception (e.g., Endler, 1975; Sarason et al., 1975; Endler and Magnusson, 1976; Magnusson and Endler, 1977), and onward, with results sometimes favoring the person and sometimes the situation, depending on the design of the research (Bem, 1972; see also Wachtel, 1973). Since then, the inclusion of potentiating environmental factors, whether life events like stressors or encounters with relevant situations, or experimental manipulations, for example, contextual “primes” that bring to mind trait-relevant content (e.g., Moskowitz, 1988; Schmit et al., 1995) has become less atypical, as researchers have examined both transient and more stable factors in observed personality responding (e.g., Chaplin et al., 1988; Murtha et al., 1996; Pervin, 2000). The stability of traits over time is of course well-argued and demonstrated (e.g., Block, 1971; Costa and McCrae, 1988; McCrae and Costa, 1990; Roberts and DelVecchio, 2000), and in conjunction with the person by situation debate, which addresses variability by context (even if just referring to contextual “primes”), the research on cross-situational variability is important and revealing about personality processes and content.

Considered differently, a question that arises is: What constitutes personality (and individual differences in personality) in the first place? It is presumably not restricted to trait dispositions. For example, more specific dispositional tendencies are presumably pertinent as well, such as the chronic individual difference of believing one is falling short of the ideal standards that a significant other holds for one, in longstanding goals with a significant other (e.g., for affection) that may have chronically gone unsatisfied, and more broadly, individual differences in chronic depression, or rejection sensitivity, or attachment style. We see these as deeply relevant to an interpersonal view of personality although such individual differences are not as broad as global trait dispositions *per se*, and the former have been examined in research on the relational self and transference (noted below). Trait dispositions, on the other hand, have not.

Beyond simply examining individual differences in personality, a central focus in conceptualizing personality as involving the relational self and stored knowledge about significant others is on illuminating what makes a person unique (Allport, 1937; Kelly, 1955; see also Higgins, 1990). Certainly, this is the thrust of George Kelly’s approach to personality. We also aim to examine, not so much what is general and global in dispositions, but rather, what is idiographic about the individual in the domain of relationships. Indeed, the content of significant-other knowledge is idiographic—that is, the features that define significant-other knowledge are varied, including assumed qualities, habits, and the like, and these features are specific to each individual and to the particular relationship—and also, by definition, stable over time. The relationship with the significant other is also unique to the person and is comprised of content that is specific to the way the individual interacts with that other, which is in turn indirectly evoked with new persons when the significant-other representation is implicitly cued. On the other hand, the process itself that is triggered when contextual cues activate such prior knowledge—the overall process by which significant-other representations are activated and used with a new person—is common and nomothetic across people (Andersen and Chen, 2002). Our approach to the relational self (and transference), which is an idiographic-nomothetic approach that captures the unique in stored knowledge and the general in process, ultimately integrates both what is stable in the self and personality, and what is variable across triggering cues, providing a more nuanced and complete view.

Still, global traits and dispositions could, indeed, be readily examined in relation to this process. Based on existing research, we assume that the process of transference is likely to be triggered and to transpire quite readily regardless of individual differences in trait dispositions. However, the content of any individual’s relational responses (those that depend on the relationship and the self in the relationship) may well vary considerably from another individual’s, based on such dispositional differences, potentially predicting differentiated affective, and motivational responses. These are empirical questions that remain open.

On this note, one might ask the question of how this relational self (and transference) research links or specifically interacts with existing structural models of personality emphasizing interpersonal traits, such as affiliation, extraversion, agreeableness, or dominance. While the current research does not speak to the exact ways in which such dispositions may emerge in transference and the relational self, such interpersonal traits have been found to be of importance in interpersonal situations (see McClelland, 1985). For instance, the need for affiliation as well as for intimacy has been shown to underlie behavioral variability in interpersonal contexts (McAdams and Constantian, 1983; McAdams, 1999), for both men and women, and is assessed mostly using the thematic apperception test (TAT). As such, correlational work shows that those high in need for intimacy are more motivated to connect, share, and communicate with others, and are inclined to focus more on communal goals (McAdams and Powers, 1981; McAdams and Constantian, 1983), in addition to making more eye contact, and smiling and laughing more (McAdams et al., 1984). Likewise, extraversion, which is part of the Big Five (and also assessed by self-report), has been associated with one’s ability to create positive social environments (Eaton and Funder, 2003). Indeed, extraverts tend to be more popular (Paunonen, 2003) and tend to have more satisfying romantic relationships (e.g., Watson et al., 2000). Relatedly, agreeableness (i.e., another Big Five trait assessed by self-report) is associated with better interpersonal adjustment among peers in adolescence (Graziano et al., 1997), more helping behavior (e.g., Graziano et al., 2007), and likeability (Nikitin and Freund, 2015). It has been linked as well to more distress in response to interpersonal conflict (Suls et al., 1998), and to preferences for tactics of de-escalation, such as negotiation rather than power assertion (Graziano et al., 1996). By contrast, trait dominance has been associated mainly with others’ perceptions of the individual as competent (as reported by fellow group members and outside observers; Anderson and Kilduff, 2009), and relatedly, dominance-conveying behaviors (assessed by independent observers) have been shown to be more commonly expressed with same-sex friends than with same- or opposite-sex strangers (Moskowitz, 1988).

With any such global trait disposition, such as these, one might predict that some of the processes revealed in the research on transference and the relational self may be more pronounced among people high in the particular interpersonal trait. Our guess is that this may not necessarily be the case, and rather, it is likely that each of these particular traits may further predict the content of the particular relational self and relationship patterns that arise, based on significant-other activation and use (e.g., in transference). This question may warrant future examination.

### Cognitive Models

One way of thinking about the notion of transference is from the perspective of George Kelly’s personal construct theory (Kelly, 1955). In this model of personality, people formulate personal constructs to represent the social environment, especially other people and themselves, by categorizing them into trait adjective terms. These personal constructs then guide individual interpretations, decisions, and actions. According to Kelly, significant others are fundamental to the constructs a person forms and stores in memory (each labeled by a trait adjective) because these constructs help the individual understand how various significant others are similar to and different from each other as well as from (and to) the self. These constructs are idiographic in nature—that is, unique to the individual. Mischel (1973) argued that such constructs are central to cognition, and are formed through basic social-learning mechanisms. By the latter extension, expectancies, learning strategies, and self-regulation are evoked by stimuli in specific situations based on what is stored in memory.

Of course, research on trait dispositions can be (and has been) conducted in cognitive terms, whether to identify the cognitive level of specificity and evaluative components of trait concepts (John et al., 1991) or to directly examine, for example, trait anxiety or neuroticism, and processes associated with each, in terms of how they are contextually cued and with what consequences (e.g., Eysenck, 2000), or to examine how trait categories influence social perception (e.g., Kenrick and Stringfield, 1980; Woike and Bender, 2009; see also Kihlstrom, 2013). Similarly, numerous researchers in clinical psychology have focused on individual differences in clinical syndromes such as phobia or major depression, and as such, they have long examined cognitive schemas of feared objects or of the self to illuminate the link between provoking stimulus cues or interpersonal situations and relevant existing knowledge (e.g., Hammen et al., 1985; Mathews and MacLeod, 1987). Still, idiographic measurement in the trait domain has remained rather atypical (although see, e.g., Lamiell, 1981; McAdams, 1996; and see also Larson and Csikszentmihalyi, 1983; Fournier et al., 2002; Conner et al., 2009).

Our model of the relational self and transference (Andersen and Chen, 2002) draws from both personal construct theory (Kelly, 1955) and Mischel’s later notion of the cognitive-affective processing system (CAPS, Mischel and Shoda, 1995), to show that individuals bearing minimal resemblance to significant others implicitly activate significant-other representations and this leads to various perceptual, affective, and behavioral consequences. In doing so, this model integrates the perspectives of psychoanalysis, behaviorism, and cognitive-behavioral approaches (Andersen and Saribay, 2006). Of course, at its core, it is a cognitive model, but conceptually it is also influenced by basic learning processes (learning theory, behaviorism) and by interpersonal psychodynamic approaches (Sullivan, 1953) that link the self to significant others and emphasize the central role of motivation and emotion. It nonetheless remains most compatible with other cognitive approaches (see, e.g., Kihlstrom, 2013).

### Personality Prototypes as Mental Representations

In contrast to a fully idiographic approach, it is worth noting that personality prototype models (Cantor and Mischel, 1977, 1979) are based on knowledge about (conceptualizations of) personality, stored in memory, and grounded largely in general, nomothetic knowledge (beliefs) about people, such as trait assumptions or notions of personality types. Personality prototypes can be defined by a trait (i.e., adjective) label, designating a main feature of an individual’s personality (and its synonyms) or by a noun label (see also Higgins and King, 1981), the latter of which is more elaborate, richer, and more distinctive in features, as in a “caricature” or stereotype of the whole of an individual’s personality (Andersen and Klatzky, 1987). As such it is used particularly efficiently in making judgments (Andersen et al., 1990). Our work expands and moves beyond such notions of personality types, overall, to what may be the richest and most distinctive of such mental representations in memory (e.g., Andersen and Cole, 1990, Studies 1 and 2)—those that designate significant others in the individual’s life (i.e., each of one’s various significant others). This should and does make these representations compelling tools for encoding. The richer and more distinctive the representations, the more accessible and likely they are to be used, a notion (about significant-other representations) that is well supported by the data, as we show (e.g., Andersen and Cole, 1990, Study 3).

Indeed, a further innovation of early work on personality prototypes was to use cognitive measures (e.g., a recognition memory paradigm) to measure, experimentally, when and how personality prototypes are applied to a new person to “go beyond the information given” about this new person (Bruner, 1957; Cantor and Mischel, 1977). In this research, these traits were conceptualized as cognitive concepts, held in memory (see also Kenrick and Stringfield, 1980; Kihlstrom, 2013), that implicitly influence the kind of personality assumptions that the individual tends to make about others. Research on transference (e.g., Andersen and Cole, 1990) adapted that experimental paradigm (Cantor and Mischel, 1977) and, in so doing, offered the first evidence that transference occurs as a social-cognitive process in everyday perception. Further, the transference effect has been replicated repeatedly, including a variety of control conditions designed to rule out alternative explanations, and measuring a variety of consequences beyond the signature biased inference/memory and evaluation effects, all of which arise based on a new person’s resemblance to a significant other (and the relationship and relational self evoked). Such consequences include relevant shifts in the motivation and goals that are pursued, behaviors that are enacted, and the sense of self experienced, along with relevant shifts in emotions (e.g., Andersen et al., 1995, 1996; Baum and Andersen, 1999; Glassman and Andersen, 1999a; Berk and Andersen, 2000, 2008; Berenson and Andersen, 2006; Reznik and Andersen, 2007; Miranda et al., 2013; Przybylinski and Andersen, 2015). Of course, a significant-other representation is an *n*-of-one representation, and hence, not a personality prototype *per se.*

## Psychoanalytic and Psychodynamic Concept of Transference

### The Psychoanalytic

Historically, the concept of *transference* has been focal in psychoanalysis (Freud, 1958), and has referred to the assumption that patients re-experience unconscious psychosexual impulses (libidinous drive) and conflicts from childhood with their analyst (Freud, 1958, 1963; see also Andersen and Glassman, 1996). Libidinous drive fuels structures of mind (id, ego, and superego), he proposed, and although he did note that “imagoes” may be formed of one’s parents, these have no causal role in the theory. Indeed, in the drive-structure model (Greenberg and Mitchell, 1983), the structures of mind and the unconscious psychosexual drive that fuels it are universal; people vary mainly in intensity of their libidinal drive. Transference, in his view, is thus fueled by libidinal impulses and processes. Freud acknowledged too that transference can transpire outside of the patient-therapist relationship, but this was far from his emphasis (Freud, 1958; see Luborsky and Crits-Christoph, 1990). Our emphasis is on social-cognitive processes in which “transference” occurs in everyday perception and interpersonal interaction, arising as an ordinary, non-defensive process, based on significant-other knowledge stored in memory that is triggered situationally by interpersonal cues. Hence, ours is a distinctively non-Freudian characterization, although we retain the term and the overall assumption that something about one’s past experience emerges in the present.

### The Psychodynamic

More specifically, our approach draws directly from that of the neo-Freudian, Harry Stack Sullivan, an interpersonal psychodynamic theorist who contradicted most of Freud’s assumptions (he dropped the entire drive-structure model, the notion of infantile psychosexual drive, and unconscious libidinal wish), focusing instead on actual interpersonal learning. Sullivan proposed the notion of *parataxic distortion*, a version of transference in which “personifications” of significant others and of the self (linked through “dynamisms” or relational dynamics) both emerge with new people (Sullivan, 1953). Personifications and dynamisms are somewhat analogous to mental representations of significant others and the relationship, respectively, and are developed through actual learning and interpersonal interactions with significant others (rather than drive). Given that Sullivan rejected assumptions about psychosexual drive made by Freud, he proposed instead basic needs for satisfaction and safety (security). Expressing one’s own perceptions, feelings, and beliefs in words with others, and also developing one’s own talents and capabilities, in each case while managing to remain connected (“integrated”) with significant others, together fulfill the former need. Security is compromised if a balance across these components of satisfaction cannot be reached. As such, the content of personifications and dynamisms includes these motivations and how they were (or were not) met with the significant other, and these are reflected in parataxic distortion with others (i.e., transference) as well. Sullivan’s assumptions about motivation are quite consistent with ours, although we have proposed other human needs as well—that is, for connection, autonomy, competency and control, comprehension/meaning, and security (Andersen et al., 1997; Andersen and Chen, 2002). Like Freud, Sullivan emphasized transference in the therapeutic context. On the other hand, he also discussed its occurrence in everyday life and did so more than Freud did. We, of course, emphasize significant-other representations and their association in memory with the self, as well as the processes by which they are brought to mind in new interpersonal encounters, affecting perception and behavior. Our approach is thus vastly closer to Sullivan’s than to Freud’s, even though we adopt Freud’s term–transference–simply because it is less cumbersome and, generally, more recognizable.

It is also worth noting that aspects of Sullivan’s interpersonal model are similar to object relations theory, as described by Melanie Klein and others, with the exception that the former emphasizes interpersonal learning and behavior as well as actual interpersonal experiences, while the latter focuses more on fantasy and libidinal drive (Klein, 1946, 1952; Greenberg and Mitchell, 1983; Grotstein, 1985). Still, like Sullivan, object relations theorists assume a notion similar to mental representations of significant others—individuals develop internalized relations with objects (significant persons) in the environment, and engage in projective identification in which these internalizations can be projected onto others. They also largely focused on transference in the therapeutic context, while not rejecting that it may arise in everyday life.

### Attachment Theory

Attachment theory is yet another framework in which interactions with significant others are thought to contribute to the development of internal working models of the self and others that are then used in subsequent relationships, influencing beliefs, memories, emotions, expectations, and behaviors about others as well as the self (Bowlby, 1973). These working models are developed from early interactions with attachment figures, reflecting expectations about the availability and responsiveness of the caregiver in times of stress, and whether or not the self is competent and worthy of love (Bowlby, 1969). A core assumption is that these working models serve as the basis for later relationships. Much research has focused on infant-caregiver interactions in the Strange Situation paradigm as well as toddler/child-caregiver interactions (e.g., Thompson, 1998, 1999), and of course, on adult attachment in romantic relationships that involve categories of secure or insecure attachment, assessed by self-report (e.g., Hazan and Shaver, 1987; Bartholomew, 1990; Griffin and Bartholomew, 1994; Pietromonaco and Barrett, 2000; Mikulincer and Shaver, 2007). While the latter function as a broad individual differences (e.g., avoidant attachment), people also have relationship-specific working models (e.g., Baldwin et al., 1996; Overall et al., 2003; Klohnen et al., 2005). Working models in the attachment framework are similar to mental representations (of self, other, and the relationship) in that they guide responding in new situations, when relevant. Hence, although our model does not originate from attachment theory, or share its precise focus, it is clearly compatible as a framework.

### In Sum

Since its inception, the concept of transference has been examined largely theoretically, rather than empirically, and thus rarely subjected to the scrutiny of science (with some exceptions, e.g., Horowitz, 1989, 1991; Luborsky and Crits-Christoph, 1990). How transference has been defined has also differed depending on the theorists involved (e.g., Greenson, 1965; Ehrenreich, 1989), with the common components tending to reflect “the experiencing of feelings, drives, attitudes, fantasies, and defenses toward a person in the present which are inappropriate to the person and are a repetition, a displacement of reaction originating in regard to significant persons of early childhood” (Greenson, 1965, p. 156). Although the conception of transference and the data we have are compatible with this definition in broad strokes, our framework and data focus on a wide variety of significant others (not only from early childhood) and highlight the precise cognitive processes that evidence suggests underlie transference. Specifically, our framework and data emphasize what is likely to trigger transference and how—that is, under what circumstances and with what consequences—leading to precise predictions that are subjected to experimental test. Such evidence emphasizes the specific nature of each particular significant other in one’s life and the relationship one has with him or her, as well as the self as experienced with that significant other, while also examining the basic cognitive processes that underlie the transference effect. We examine the cues in an interpersonal situation that trigger transference, the specific processes that prompt it, and its precise consequences that arise via what knowledge is stored in memory.

## Evidence Supporting Transference and Relational Self

### A Word on Methods

Research on transference typically uses a two-session paradigm. In the first session, participants name and describe at least one significant other by listing an equal number of positive and negative sentences (two to six words each)—about the significant other’s interests, likes, attitudes, beliefs, tendencies, or specific behaviors or styles (e.g., likes to think about politics, plays the flute, is even-tempered)—and then rank-ordering the sentences based on their descriptiveness of this significant other. Weeks later, participants return for a supposedly unrelated experiment, and are for example, randomly assigned to a condition in which they learn about a new person who is described using some of the features the participant listed in the first session about their significant other intermixed with filler items that are indicated as being irrelevant to the significant other, or a new person who does not resemble a significant other at all (e.g., Andersen and Baum, 1994; Andersen et al., 1996; Berk and Andersen, 2000; Berenson and Andersen, 2006). Hence, in one condition the new person bears a subtle resemblance to the significant other. In a yoked control condition, significant-other descriptions are instead drawn from those that another participant listed about his or her significant other. This one-to-one yoking of a participant in the control condition with one in the transference condition ensures that those in the resemblance and control conditions are presented with exactly the same descriptions. This allows us to control for content of the features used and thus rule out the possibility that descriptions of any significant other can trigger the transference process. After being presented with descriptions about the new person, participants complete various dependent measures.

It is worth noting as well that we have also made use of a fully within-subjects design in which the participant learns about various new persons, one of whom resembles their own significant other, while the other new persons do not, allowing for the examination of shifts in the same participant’s responses as a function of triggering cues (e.g., Andersen and Cole, 1990; Andersen et al., 1995; Glassman and Andersen, 1999b). In addition, we often cross such within-participant manipulations with between-subject factors in a mixed model design (e.g., Chen et al., 1999; Przybylinski and Andersen, 2013, 2015).

### Signature Cognitive and Evaluative Effects

#### Inference and Memory

Early work on transference has assessed activation of a significant-other representation and its use via a recognition-memory paradigm assessing what one remembers about a new person and the tendency to “fill in the blanks” about him or her using the significant-other information stored in memory (adapted from Cantor and Mischel, 1977). Research has demonstrated that after learning about a new person who exhibits some subtle similarity to a significant other, individuals will tend to assume that the new person possesses other features of their significant other. That is, they report higher confidence in having learned specific features about the new person that they in fact did not—features relevant to significant-other knowledge (e.g., Andersen and Cole, 1990; Andersen and Baum, 1994; Andersen et al., 1995; Baum and Andersen, 1999; Berenson and Andersen, 2006; Saribay and Andersen, 2007; Przybylinski and Andersen, 2013, 2015). The individual remembers the new person in a manner colored by the stored significant-other knowledge, based on subtle resemblance to the significant other (versus to a yoked participant’s significant other). For instance, if an individual finds out that a new person likes to knit and it so happens that the individual’s own sister likes to knit and is also very self-confident, the individual is more likely to incorrectly remember having learned that the new person is actually very self-confident.

In this research, resemblance to a significant other is usually constructed as a small number of significant-other based features embedded among distractor cues (irrelevant features), which makes the triggering/cueing process relatively implicit in all of our experiments. Accordingly, the effect has been found even when significant-other features are presented subliminally (Glassman and Andersen, 1999a), and thus outside of conscious awareness. The transference effect occurs effortlessly, and cannot be controlled easily—that is, it is automatic (see Andersen et al., 2007)—and also persists over time (Glassman and Andersen, 1999b). Moreover, the effect arises even when the significant-other representation is activated based on subtle facial resemblance to a new person. That is, individuals made inferences about the new person consistent with knowledge of their significant other after being presented with a photograph, allegedly of the new person, which they had previously rated (in a prior session) as resembling their significant other (Kraus and Chen, 2010).

To rule out alternative explanations of such effects, potential experimental confounds have been carefully examined. For instance, these effects could possibly be accounted for by the fact that participants themselves generated their own significant-other features used to describe the new person in a previous session (Greenwald and Banaji, 1989), but did not generate such features for the yoked-control condition. Since memory tends to be better for self-generated materials, a control condition is used to include descriptions that the participant also self-generated, but instead to describe a social category the person tends to use (Andersen and Cole, 1990), or a non-significant other in the person’s life (e.g., Andersen et al., 1995; Glassman and Andersen, 1999b), or in some cases, no one person at all (the no representation condition, e.g., Przybylinski and Andersen, 2013, 2015). Such control conditions address self-generation effects. The inference and memory effect, in sum, is stronger for representations of a significant other than for other self-generated information and for social categories the individual tends to use. Hence, the effect cannot be reduced to simple social categorization effects (e.g., stereotyping) or to self-generation.

Much research shows that transference is quite pervasive and occurs even averaging across individual differences in relationships. Indeed, the transference effect, as indexed by this signature cognitive measure, occurs regardless of whether the individual views the significant other positively or negatively (e.g., Andersen and Baum, 1994; Andersen et al., 1996; Hinkley and Andersen, 1996). It also emerges independent of the self-discrepancy the individual may have from a parent’s standpoint (when the parent is the significant other), for example, if the individual has fallen short of the parent’s standards (Reznik and Andersen, 2007), and regardless of psychological and physical abuse by a parent (as the significant other) while growing up (Berenson and Andersen, 2006), chronically dissatisfied affection goals with the significant other (Berk and Andersen, 2008), and of depressive symptomatology (Andersen and Miranda, 2006; Miranda et al., 2013). Unsurprisingly, other affective and motivational consequences also arise in this process (noted below), usually as a function of the relationship that is indirectly activated when the significant-other representation is cued.

Finally, as further evidence that the transference process does in fact emerge quite automatically, evidence shows that the transference process is moderated by variables that are known to moderate other automatic processes. Research in other labs has shown that transference is more likely to occur if the individual is experiencing a circadian rhythm mismatch (it is the wrong time of day for him/her, Kruglanski and Pierro, 2008), or is high in need for closure (Pierro and Kruglanski, 2008), or is not inclined to engage in careful assessment (Pierro et al., 2009).

#### Evaluation

Individuals evaluate people and objects quickly and relatively automatically (e.g., Bargh et al., 1996). Such snap judgments can be influenced by significant-other representations. That is, when a significant-other representation is activated and applied to a new person in transference, the way one evaluates the significant other is also implicitly evoked and applied to the new person, and the person is evaluated as the significant other is—that is, positively or negatively. This significant-other based evaluation is grounded in the notion of schema-triggered affect (Fiske and Pavelchak, 1986), as it arises based on the triggering of a significant-other representation, and this effect is considered another signature effect of transference. Indeed, a new person will be evaluated more positively in self-reported Likert ratings if he or she minimally resembles an individual’s own positive significant other versus a yoked participant’s positive significant other (Baum and Andersen, 1999) or versus an individual’s own negative significant other (Andersen and Baum, 1994; Andersen et al., 1996; Berk and Andersen, 2000). As noted, this effect also occurs across a wide variety of relationships, for example, when the significant other is a parent and the individual believes he or she falls short of the parent’s standards (Reznik and Andersen, 2007; see Higgins, 1987), or if the individual was abused by the parent (shown in facial expressions, Berenson and Andersen,2006).

Indeed, snap judgments can also be triggered by minimal facial resemblance in a new person, as shown by research from two other labs—for instance, when the new person is depicted using a photograph that was previously rated by the participant as similar (versus not) to a loved significant other (Kraus and Chen, 2010), he/she is rated more positively. Along these lines, when the new person is depicted using a photograph that was created by morphing the face of a loved significant-other with another person’s face (Günaydin et al., 2012), the individuals tended to indicate (by saying “yes” versus “no”) that the new person possessed certain positive traits, such as trustworthiness or intelligence. Moreover, automatic positive evaluation of a new person, based on his or her significant-other resemblance, is enhanced when one’s own mortality is made salient (Cox et al., 2008, Study 5). That is, research in yet another lab has shown that a parent-resembling new person is evaluated more positively, especially if one has just thought about one’s own death (versus about extreme pain; Cox et al., 2008), suggesting that transference involving a loved significant other is more likely in the face of death threat and may thus serve terror management functions and serve existential needs (see Przybylinski and Andersen, 2015).

Indeed, when the significant other is regarded positively, an immediate positive emotional response should be elicited, and there is evidence on the individual’s own facial expression of affect to support this. When the new person is similar to a positive rather than a negative significant other, more positive facial affect is expressed (as unobtrusively recorded), and this occurs quite quickly—that is, as one reads the relevant features presented about the new person (Andersen et al., 1996). The quick emergence of this emotional response suggests automatic evaluation of the new person (Bargh et al., 1996), arising in the transference process, based on evaluation of the significant other. As noted, this effect is even evident when the significant other is an abusive parent from one’s childhood (Berenson and Andersen, 2006). People often denote that they love their parents independent of having negative or dangerous experiences with them, and these positive feelings are elicited relatively immediately in transference in the form of positive facial affect (Berenson and Andersen, 2006).

### Relationship Effects

#### Expectancies

Intermixed with the information one has about significant others, is information regarding how each significant other relates to and behaves toward the individual, and this knowledge should be applied to new people resembling these significant others in transference. Indeed, research shows that the acceptance or rejection one expects from a significant other is activated and applied to the new person in transference. When a new person resembling a positive versus negative significant other is encountered, the new person is expected to be more accepting and less rejecting, an effect that does not hold in the control condition (Andersen et al., 1996; Berk and Andersen, 2000). Thus, the individual anticipates being liked or disliked by the person based on expectations he or she has of the significant other, and this occurs across individual differences, such as one’s self-discrepancy from the parent’s perspective (Reznik and Andersen, 2007), whether or not the significant other has typically shown the level of affection one has desired (Berk and Andersen, 2008), whether or not one has been rejected by a loved significant other (Miranda et al., 2013), and whether or not a parent was abusive in the past (Berenson and Andersen, 2006).

Beyond the transference context, relationships (as stored in memory) are known to be linked with overall expectations of rejection or acceptance (e.g., Baldwin and Sinclair, 1996). Knowledge of how accepting or rejecting a significant other is, is stored in memory, and such expectancies are readily activated, whether in relationships or based on priming of the relationship (e.g., Baldwin et al., 1990; Miranda et al., 2013); this is especially so if the individual tends to be rejection-sensitive (Downey and Feldman, 1996).

#### Interpersonal Behavior

Another important component of significant-other relationships is behavior. The typical behaviors engaged in with the significant other should also be activated in transference and enacted with the new person, evoking behavioral confirmation—or a self-fulfilling prophecy. The new person should then enact behaviors the individual expects of him or her. Indeed, individuals having a phone conversation with a naïve stranger who was made to resemble a positive (or negative) significant other—or not—evoked expected behaviors from the new person (Berk and Andersen, 2000). The new person’s part of the conversation, as assessed by the ratings of independent judges who were blind to condition, connoted the expected positive or negative affect, based on how positively or negatively the significant other was regarded, and this effect was not evident when the new person did not resemble a significant other. Some research suggests that this effect occurs without specific intention and thus individuals do not consciously attempt to elicit the expected behavior (e.g., Chen and Bargh, 1997). We assume this is what happens in transference. In a familiar example, an individual may unknowingly respond to a new person as though he is a past romantic partner without realizing that he or she is doing this, and in turn the new person may start to behave as the romantic partner would.

Furthermore, individuals in transference will also, under some circumstances, engage in behaviors designed to solicit liking and positive responding from the new person despite a troubled relationship with the significant other. That is, when a new person resembled a well-regarded significant other who commonly failed to satisfy one’s goals for affection, individuals not only became more hostile as a result, but this hostility was linked to persisting longer on a behavioral task said to increase positive response from others (Berk and Andersen, 2008). Thus, behaviors done to achieve a particular goal one has with the significant other are evoked and enacted in transference.

#### Motivation and Goals

The motives and goals held with significant others are stored with significant-other knowledge in memory, and evidence shows that they are, indeed, brought to mind and applied when significant-other knowledge is evoked. In transference, goals to be close to positive significant others are frequently activated and pursued with a new person in the context of transference. That is, individuals are motivated to approach and to be disclosing toward a new person who bears minimal resemblance to a positive significant other and to avoid closeness when the new person is similar to a negative significant other (Andersen et al., 1996; Berk and Andersen, 2000). As further evidence of this, behavioral approach motivation has also been shown to emerge in a positive transference. That is, individuals moved their chairs closer to where they were told the new person would sit for an upcoming interaction if this new person resembled the positive significant other versus not (Kraus et al., 2010).

However, this behavior is also relationship-specific. For example, when a loved significant other has not met one’s goals for love and affection—the goal is unsatisfied—this knowledge emerges in transference (Berk and Andersen, 2008). When such a significant other is evoked by resemblance to him or her in a new person, the usual positive affect evoked by transference is disrupted and the individual shows a decreased motivation to be close and disclosing to the new person, even showing increased hostility. Interestingly, when the significant other in this study was a relative (suggesting that the relationship is not likely to be ended), the hostility expressed by the individual was positively related to increased enactment of explicit behaviors that would help attain acceptance and liking from the new person (Berk and Andersen, 2008). The goal that had not been fulfilled with the significant other was pursued in transference, even as the individual became more hostile and even if these mixed messages—expressing hostility yet also seeking affection—may prove particularly confusing and frustrating to the new person.

Beyond this, transference not only prompts goal activation and goal pursuit—when mental representations of significant others are activated—but also specifies both the *how* and *why* of goal pursuit based on the activated relationship. That is, activation of significant-other knowledge shapes both the overall goal to be sought, as the *why* of goal pursuit (its higher-order goal), and the subgoal to be selected, as the *how* of goal pursuit, or the means of pursuing that goal (Ahn and Andersen, 2016). Such evidence attests to the richness of the transference concept in its implications for motivation and goals.

Even achievement goals can be brought to the fore in transference. When a significant other holds such a goal for the individual and knowledge of that significant other is implicitly activated in transference, the individual will actively pursue the achievement goal in behavioral terms at that moment (Xu and Andersen, 2014). Moreover, such goals from a prior significant-other relationship can be triggered and enacted even with a current romantic partner (versus only with new persons). Thus in transference, achievement goals from a prior relationship may be suddenly pursued with a current romantic partner, even if this goal is potentially disruptive to the current relationship (Xu and Andersen, 2014).

Finally, research outside the domain of transference has shown other goals can also be evoked when a significant-other representation is activated. For instance, priming a significant-other representation increases the pursuit of goals that one has with the significant other—such as competition, achievement, and helping goals (Fitzsimons and Bargh, 2003; Shah, 2003a,b) as well as attachment-style congruent goals when a significant other with whom one is securely or insecurely attached is primed (Gillath et al., 2006).

Moreover, research from other labs has again shown that while significant others do affect the kinds of goals that are activated and pursued, goals that are activated at the moment also can, in turn, have implications for social perception and social categorization. When a goal has been primed, individuals spontaneously bring to mind the individuals in their life that are instrumental to the goal—useful for pursuit of the goal—or not. For instance, after a goal is primed, individuals make more memory errors between individuals within categories of “instrumental” and “non-instrumental,” respectively, suggesting that social categorization also depends on the kinds of goals that are active (Fitzsimons and Shah, 2009). On the other hand, activating a significant other does not always foster active behavioral goal pursuit of the most relevant goals in that relationship and may even undermine motivation, since people often tend to “outsource” goal pursuit plans to significant others who are supportive of those goals (Fitzsimons and Finkel, 2011). Moreover, being subliminally exposed to the name of a significant other who is controlling can elicit reactance and oppositional behavior (Chartrand et al., 2007), depending on the nature of the relationship.

#### Some Effects of the Self in Relation to the Other

Likewise, individuals tend to form a version of the self in the context of a particular relationship, as the self is typically experienced with each specific significant other, and this relational self should be evoked in transference. Indeed, in transference, an individual’s sense of self should parallel the version of the self that is experienced with the relevant significant other. For example, one may be especially gentle and supportive toward one’s wife, and yet one may be assertive and seek power amongst co-workers. These various ways of perceiving the self and behaving–for example, being both gentle and assertive–can each constitute the self; however, in line with Mischel’s IF–THEN theory (Mischel and Shoda, 1995), they differ based on the interpersonal context in which they are activated. When the significant other representation is activated, the corresponding self is activated and enacted, as well (Andersen and Chen, 2002).

Indeed, there is evidence to support this. Adjusting for one’s self-definition at pretest, the “relational self” with the specific significant other is experienced with the new person, independent of whether the significant other is regarded positively or negatively (Hinkley and Andersen, 1996). The knowledge one has of the self with the significant other, as well as its valence, enters the working self-concept when the significant-other representation is evoked. This effect can also be provoked by facial resemblance to a significant other in a new person. When presented with a photo of a new person whom individuals had previously rated as resembling a significant other in an earlier session, individuals described themselves more as the person they are when with the significant other (Kraus and Chen, 2010).

Activation of the relational self has also been shown to indirectly activate automatic self-verification processes in transference. Research from another lab showed that upon learning about a significant-other resembling person (versus not), individuals end up rating themselves in a manner that better reflects their desired self (how they would like to be viewed) on self-attributes most important to the relational self (Kraus and Chen, 2009). In the absence of significant-other resemblance, by contrast, individuals are more likely to self-enhance using a wide variety of attributes. In fact, when a significant-other representation is activated, individuals will also describe themselves (e.g., how athletic or artistic they are) to a new person resembling the significant other in such a way as to receive self-verification for important aspects of the activated relational self (Kraus and Chen, 2014).

Along the same lines, one’s sense of self-worth is also dependent in part on the relational self that is active at the moment. Contingencies of self-worth that are experienced with a significant other (e.g., Crocker and Wolfe, 2001; Horberg and Chen, 2010) are activated when that significant-other representation is implicitly activated in transference. Research from another lab showed that when a significant other with whom individuals wanted to be close to was implicitly activated, individuals were more likely to stake their self-esteem on performance in those domains in which the significant other wanted them to do well (Horberg and Chen, 2010). That is, individuals’ sense of self-worth and the degree to which they had thoughts about failure were affected by perceived success or lack thereof on performance in that particular domain.

Relational selves can also help affirm one’s overall sense of self when aspects of relational selves are deemed important. For example, research on self-affirmation and relational selves from another lab suggests that individuals who see relational self-aspects as particularly important to their identity, can readily maintain a positive sense of self in the face of threat (e.g., negative feedback on an aptitude test) by focusing on these particular aspects of the self (Chen and Boucher, 2008), and in so doing, protect their self-esteem. That is, individuals threatened by bogus negative feedback showed a heightened tendency to characterize themselves in relational terms in a self-description task (Study 1) and to evaluate positively the letters in their own names (versus other letters, Study 2), suggesting positive implicit self-esteem (Chen and Boucher, 2008).

Cultural differences also sway how the self is contextually experienced and perceived in relation to different significant others. For instance, research has shown that Asian Americans show more cross-situational inconsistency across different relationships than do European Americans, and yet they do maintain consistency in self-descriptions—within each relationship—over time (an across-time consistency often found in dispositional research, writ large, English and Chen, 2007). Further, among European Americans, but not Asian Americans, inconsistency in individuals’ self-perceived traits across different relationships has been shown to be associated with reduced feelings of authenticity and relationship quality. Both groups, however, experience lower levels of authenticity and relationship satisfaction based on perceived inconsistency in the same relationship over time (English and Chen, 2011). In short, this research suggests that Asian Americans may be even more likely to experience the self on an if-then basis, even though we know European Americans also show such cross-situational consistency, as noted.

In addition, evidence stemming from other labs does show that the attachment system is triggered in transference (e.g., Cox et al., 2008, Study 5) and that attachment style and working models clearly emerge in transference (e.g., Brumbaugh and Fraley, 2006, 2007). In the classic transference paradigm, manipulated resemblance to a prior romantic partner led individuals to apply their attachment style with a past romantic partner (the prior significant other) to a potential dating partner, as reflected in self-reported anxiety and avoidance (Brumbaugh and Fraley, 2006). Likewise, such manipulated resemblance led the overall attachment style to arise in relation to potential new friends (again in anxiety and depression, Brumbaugh and Fraley, 2007). Beyond this, other research indicates that manipulated mortality salience (thoughts of death) makes transference more pronounced in relation to a new person (based on manipulated resemblance to a parent), in terms of self-reported liking and also behaviors such as arranging for less physical distance (more physical closeness) with the new person (Cox et al., 2008, Study 5), increasing the relevance of the parent as a secure base, and hence, the new person as a safe haven.

Hence, the relational self that is active at any particular time is clearly dependent on important situational cues—that is, whether or not a new person bears a resemblance to the significant other. IF significant-other resembling cues are present in the situation, THEN the significant-other representation will be evoked, and the self-with-that-significant-other will become the functioning self-concept at that time.

#### Self-Regulation

In addition to shifts in the working self-concept as a function of the implicit activation of a significant-other representation, contextual self-regulation is also evoked in transference. One example of this is when negative self-with-significant-other features enter the operative self-concept in transference, and thus pose a threat to the self, a self-defensive response should be elicited—as is common in response to other threats (see Greenberg and Pyszczynski, 1985; Steele, 1988). Indeed, when a significant other who is associated with a disliked version of the self is evoked (Hinkley and Andersen, 1996; Reznik and Andersen, 2007), the negative self-with-other features that have been evoked should lead to the positive features that are not a part of the relational self to enter into the working self-concept, shielding one’s image of the self from threat, and this is in fact the case (Hinkley and Andersen, 1996; Reznik and Andersen, 2007).

Similarly, the individual may, under some conditions, come to protect the significant other in transference. This kind of regulation may occur because it is favorable for people to believe that their significant others are generally loving and good, despite their faults. Thus, significant-other faults are often transformed into charming quirks and even virtues (e.g., Murray and Holmes, 1993). In transference, this process may occur relatively automatically if it tends to occur consistently in the relationship over time. Indeed, as previously noted, immediate facial affect expressed in transference connotes the general feelings related to the significant other. Furthermore, when negative features of a positive significant other are encountered, participants express even more positive immediate facial expressions than when they encounter positive features, an effect not evident in the control condition (Andersen et al., 1996). Thus, it appears that facial affect transforms the valence of the feature from negative to positive, so as to parallel the general positive affect related to the significant other. This implies that self-regulation of this kind is evoked because encountering a negative feature of a liked or loved significant other in a new person may threaten the positive regard one has for the significant other.

Such self-regulation could in principle extend to a significant other’s negative emotions or behaviors when they are expressed by a new person, as well. In particular, for example, if a significant other has psychologically and physically maltreated an individual in the past, a cue that may suggest rising tension from this significant other should be especially negative; however, it should still elicit self-regulation from the individual. Hence, cues like anger from the significant other are likely to signal impending abuse and may prompt self-regulation in the individual to shield him or her from its consequences. Research has tested whether or not new people in transference can trigger similar self-regulation processes. For example, research tested how individuals abused as children by a parent (or not) respond in transference when the new person displays the pattern typical prior to abuse, such as becoming more irritable when awaiting the interaction (Berenson and Andersen, 2006).

Although abused individuals displayed immediate positive facial affect in transference, they expressed negative evaluations of the new person compared to a control condition—they expected rejection from him or her, were indifferent to whether or not the new person liked them, and experienced significantly more negative mood. When they were told that the new person was becoming irritable and angry, however, those in transference relative to the yoked control condition exhibited more positive facial affect regardless of abuse history, presumably to maintain general positive regard for the significant other. Previously abused participants showed comparable levels of positive facial affect as non-abused participants after encountering this cue. Such a regulatory response aimed at protecting the other may not be wise if the new person who is similar to the abusive significant other is also abusive; that is, abuse could be perpetuated in a new relationship. Interestingly, abused participants exhibited much less negative affect when the new person resembled their parent and was also acting angry and irritable compared to participants in all other conditions, a kind of apathy that is referred to as “emotional numbing” in the abuse literature.

In addition, individuals who possess certain emotional vulnerabilities may be particularly likely to experience negative affect when a loved, but rejecting significant other is activated in transference. For example, dysphoric and non-dysphoric college students who expected to meet a new person resembling a loved, but sometimes rejecting, significant other were asked to describe themselves by completing sentences to assess their working self-concept (as in Hinkley and Andersen, 1996). Dysphoric individuals described themselves in terms that were rated by judges as more rejecting (Miranda et al., 2013). They also experienced an increase in negative affect. However, when the new person resembled a yoked significant other, or even a disliked significant other, this effect did not occur. In fact, dysphoric individuals showed a decrease in depressive mood when a disliked significant other was brought to mind compared to a yoked participant’s significant other. Non-dysphoric individuals did not experience such shifts in mood following the activation of any of the significant-other representations.

Another way of thinking about self-regulation and transference is to consider how an individual may experience having a self-discrepancy (Higgins, 1987) from the perspective of a parent, when falling short of the standards held by that parent, and the emotional vulnerability this entails. Indeed, implicit activation of a parental representation in transference should indirectly activate one’s sense of self, as well as the parent’s standards for the self, which may not be the same (Reznik and Andersen, 2007). Hence, activating a parental representation may evoke a self-discrepancy (e.g., Higgins, 1987) in transference, if such a discrepancy exists in the relationship with the parent. A discrepancy between one’s actual self and one’s ideal self (who one could ideally be) or one’s ought self (who one should be) prompts feelings of dejection (as the actual-ideal discrepancy is activated) or feelings of agitation (as the actual-ought discrepancy is activated). These precise feelings thus arise in transference, when relevant to the relationship.

However, when significant-other cues are encountered in the new person that directly bring to mind the standard a parent holds for oneself (i.e., when the new person emphasizes hopes for or obligations for new friends), the regulatory functions of the standards activated (i.e., according to self-regulatory focus theory; Higgins, 1997) should be prompted based on transference. That is, ideal standards (or hopes) are relevant to a promotion focus—a focus on seeking out potential gains, while ought standards (or obligations) are relevant to a prevention focus—a focus on threat to avoid losses. Feelings of agitation should be exacerbated when in a state of prevention focus, whereas a promotion focus should decrease feelings of dejection, and this is what evidence has shown. Individuals in transference with an actual-ideal discrepancy from their parent’s standpoint who were presented with a cue bringing to mind the parent’s ideals showed evidence of promotion focus that reduced feelings of dejection while a similar cue presented to individuals with an actual-ought discrepancy did not facilitate a reduction of agitation-related feelings in transference (Reznik and Andersen, 2007).

Other problematic inconsistencies that exist in a significant-other relationship are also activated in transference. For instance, a discrepancy in goals one has with a significant other, such as an unsatisfied goal for love and affection, as noted, is activated when the significant other is (Berk and Andersen, 2008). This leads individuals to experience feelings of hostility toward the new person, while still attempting to satisfy the activated goal. That is, when the representation of the significant other with whom they have such a goal discrepancy is activated in transference, individuals persist on a task designed to elicit liking from the new person. Similarly, dysphoric individuals who have sometimes been rejected by loved significant others, and thus have experienced a discrepancy in their relationship regarding love and affection, report increased depressed mood when such a significant other is activated (Miranda et al., 2013). They also describe themselves in a manner conveying rejection in this context.

Indeed, a different way of viewing the matter of self-regulation with respect to transference and the relational self is to ask whether or not this relatively automatic process can ever be intentionally short-circuited. When the transference process perpetuates suffering by triggering interpersonal problems, individuals may have good reason to want to prevent it. On the other hand, its automaticity—that is, the notion that it arises without effort, intention, or awareness (see Andersen et al., 2005, 2007)—should make it difficult or impossible to control (Przybylinski and Andersen, 2013). Evidence suggests that this is so. However, it can be controlled when people make use of an intentional strategy that can itself be automatized, which can in fact be effective in eliminating unwanted inferential and memory biases (Przybylinski and Andersen, 2013).

#### In Sum

Taken together, this research convincingly demonstrates how the self, as experienced from moment-to-moment, can vary as a function of situational triggering cues that activate and bring to mind stored knowledge of significant others. When such knowledge is activated, it indirectly activates the specific relational self, as well as the relationship with a particular significant other. This results in shifts in judgments, memory, evaluations, goals and motivations, as well as emotional state, and perceptions of the self, based on this activated stored knowledge. In turn, how one responds to a new person at the moment will also depend on the nature of the significant other that is activated in the moment. The effects tend to be of moderate effect size and are well replicated (e.g., Miranda et al., 2013; Przybylinski and Andersen, 2013, 2015). In this sense, the evidence demonstrates the cross-situational variability of the person in the domain of interpersonal relationships as a function of triggers in the current situation.

While we do not assume that everything about personality is interpersonal, the fact that significant-other representations have been shown to be activated quite automatically, based on incidental contextual cues, implies that even when the individual is consciously focused on other things or enacting a common routine exchange with another person, the process is still likely to unfold, at least in subtle ways. Of course, if the individual is anticipating further interaction with the person or for other reasons is more engaged, self-relevant expectations, emotions, and motivations should be more likely. In this respect, there are likely to be boundary conditions on the more emotionally laden phenomena we have observed. In addition, as previously noted, there may be circumstances under which the transference process is particularly likely to occur such as when one is experiencing a circadian rhythm mismatch (Kruglanski and Pierro, 2008), is high in need for closure (Pierro and Kruglanski, 2008), or low in assessment orientation (Pierro et al., 2009), or when one’s mortality has been threatened in some manner (Cox et al., 2008).

Finally, although we do not focus exclusively on within-person designs that directly examine variability in responses within the individual across contexts—nor focus on stability in the transference effect over time (longitudinally)—we have shown that the transference effect, as provoked (or not) by initial situational (person) cues and that this persists at least over a 2- to 3-week period concerning that new person (Glassman and Andersen, 1999b). Moreover, even when we use a between-subjects design, this still does involve long-standing (and relatively stable) stored significant-other knowledge. The experimental designs are also carefully controlled, by manipulating whether or not the individual’s own significant other is cued (i.e., exposure to differing interpersonal situations), in order to assess how responses then vary.

## Future Directions

The complex, situation-dependent ways of responding we have empirically shown to occur in transference provide support for a relational IF–THEN conceptualization of personality, and call for future research that could clarify the conditions of behavior and personality, based on situational cues. First, significant others are often not thought of as merely “positive” or “negative,” but rather possess varying degrees of ambivalence that have specific significance for one’s experience when these significant-other representations are activated. Such ambivalence, as it may be triggered situationally by transference, is in need of further testing beyond the activation of standards that the significant other holds and past abuse by that significant other.

Considerably more research on these topics is necessary, particularly that which focuses on emotion and emotion regulation in transference. For instance, it is now well-known that, although significant others may be loved, they may still be associated with painful emotions and this suffering can be perpetuated in transference. Conceptually, it is of central importance to ascertain whether or not or precisely how people may prevent the processes of transference (beyond just memory and inferences, as in Przybylinski and Andersen, 2013) from transpiring when triggering cues are present in a situation. The ability for one to regulate a relatively automatic way of responding evoked implicitly and without intention so as to not be affected by it is worth further consideration.

In addition, examining the conditions in which positive interpersonal results may come about in transference, as opposed to just negative ones, would be important to understanding the way positive interpersonal responses are related to representations of significant others. For instance, in some circumstances individuals should not only make certain mistakes in interpreting the new person, but also may pay special attention to him or her and be especially motivated to understand him or her, such as through empathy. These consequences of transference have not been adequately explored.

## Conclusion

To conclude, our model, known as the relational self, integrates the self and personality. We draw in part on Mischel’s (1968) notion of cross-situational variability in behavior, and also show consistency within individuals based in the cognitive-affective processing system and their personality signature (Mischel and Shoda, 1995; Metcalfe and Mischel, 1999). Our model shows an intrinsic link between the situation and an individual’s responses in the situation—including emotion, expectancy, and the experience of the self—that is grounded in relationships with significant others. We argue that significant-other representations can be individually activated at any time as a function of situational cues, leading to the indirect activation of the self when with the significant other, as well as the relationship with the significant other. These activated representations affect interpersonal behavior and other responses in predictable ways.

We argue that the precise responses in transference are specific to the person and to the relationship with the significant other that is evoked, even though they are stable over time—that is, when similar cues are present in the environment, similar responses should be evoked. In this way, the self and personality are, at any given moment, dependent on cues in the situation as well as on the chronic accessibility of significant-other representations. Thus, significant-other knowledge stored in memory that may be activated is stable over time, as is the version of the self one is with that other. Thus, transference occurs on an IF–THEN basis—IF one encounters someone who is similar to a significant other, THEN one becomes the version of the self when with that significant other. Indeed, this process transpires in individualistic cultures like the U.S.—where the research was conducted—as well as across genders, and among individuals not specifically preselected as allocating special attention to relationships.

Overall, the contributions of Mischel and his colleagues—both conceptual and empirical—facilitate an important shift in the field of personality. Researchers are increasingly embracing the notion that behavioral variability is fundamental to personality, and are embracing ways to test the complexity of that variability, versus regarding it as merely error variance. This can enrich our understanding of individuals greatly. As a relational IF–THEN framework, our model reflects this perspective in personality theory, and can be considered one of various IF–THEN models of personality to emerge in the last decade or so, such as those reconceptualizing CAUs (Mischel and Shoda, 1995) as “schemas” or organized knowledge structures similarly brought to the fore in relevant situations, thus allowing for situation-specific interpretive tendencies (Cervone, 2004; Cervone and Batooszek, 2013).

Our work shows the important role significant others play in understanding personality, especially affect, expectations, behavior, and how the self is experienced from one situation to the next. It also integrates ideas from psychodynamic theory, as well as those from social cognition and learning theory. Drawing on Mischel’s insights, the model combines diverse areas of psychology to map the complexity of personality, by integrating an individual’s interpersonal history with the present situation, and highlighting why an individual’s variability by context reflects essential aspects of the person.

## Conflict of Interest Statement

The authors declare that the research was conducted in the absence of any commercial or financial relationships that could be construed as a potential conflict of interest.
